# Differential tolerance to heat stress of young leaves compared to mature leaves of whole plants relate to differential transcriptomes involved in metabolic adaptations to stress

**DOI:** 10.1093/aobpla/plac024

**Published:** 2022-06-21

**Authors:** Qingyuan Xiang, Bala Rathinasabapathi

**Affiliations:** Horticultural Sciences Department, University of Florida, Gainesville, FL 32611, USA; Horticultural Sciences Department, University of Florida, Gainesville, FL 32611, USA

**Keywords:** *Arabidopsis thaliana*, heat shock protein, leaf development, pectin lyase-like superfamily protein, RNA sequencing

## Abstract

Plants respond to heat shock by regulating gene expression. While transcriptomic changes in response to heat stress are well studied, it is not known whether young and old leaves reprogram transcription differently upon stress. When whole plants of *Arabidopsis thaliana* were subjected to heat shock, young leaves were affected significantly less than older leaves based on measurements of tissue damage. To identify quantitative changes to transcriptomes between young and old leaves upon heat stress, we used RNA sequencing on young and old leaves from plants exposed to control and heat stress at 42 °C for 1 h and 10 h. A total of 6472 differentially expressed genes between young and old leaf were identified under control condition, and 9126 and 6891 under 1 h and 10 h heat stress, respectively. Analyses of differentially expressed transcripts led to the identification of multiple functional clusters of genes that may have potential roles in the increased heat tolerance of young leaves including higher level of expression in young leaves of genes encoding chaperones, heat shock proteins and proteins known in oxidative stress resistance. Differential levels of transcripts for genes implicated in pectin metabolism, cutin and wax biosynthesis, pentose and glucuronate interconversions, cellulose degradation, indole glucosinolate metabolism and RNA splicing between young and old leaves under heat stress suggest that cell wall remodelling, cuticular wax synthesis and carbohydrate modifications impacted by alternative splicing may also have roles in the improved heat stress tolerance of young leaves.

## Teaser

This study on the model plant *Arabidopsis*reveals that when whole plants are exposed to severe heat stress young leaves are affected less than older leaves. Quantitative evaluation of transcripts showed that young leaves differentially express genes related to heat shock proteins, chaperones, plant growth regulators, pectin metabolism, cutin and wax biosynthesis, pentose and glucuronate interconversions, cellulose degradation, glucosinolate metabolism and RNA splicing. These results have implications for identifying target genes for improving heat stress tolerance in plants.

## Introduction

High-temperature stress limits growth and development of plants. Heat stress negatively affects multiple functions of plant cells triggering a complex network of acclimation processes (Wang *et al.* 2004; Kotak *et al.* 2007; Richter *et al.* 2010). These responses could differ between different developmental stages and stress levels (Knight and Knight 2001; Mittler 2002; Gu *et al.* 2012). High temperatures are known to influence cell permeability and ion transport through altering membrane fluidity (Sangwan *et al.* 2002). Reactive oxygen species (ROS) rapidly accumulate within cells as a result of injury from heat stress and this might lead to programmed cell death (Breusegem *et al.* 2006; Vacca 2006; Vacca *et al.* 2007). And high temperatures can also cause enzyme inactivation and protein denaturation, leading to imbalanced metabolic pathways (Waters *et al.* 1996; Saibil 2013; Waters 2013). Damages to photosynthetic efficiency under temperatures above optimal levels could decrease crop productivity (Allakhverdiev *et al.* 2008; Bita and Gerats 2013). Reprogramming of gene expression to increase protein diversity and adaptation is required for plant responses to adverse conditions.

Heat stress during reproductive stage of plants is highly detrimental to fruit yield and hence has been thoroughly studied. However, plant acclimation to heat stress at the vegetative stages is poorly known. A study in *Zea mays* measuring photosynthetic parameters and membrane integrity showed that under heat stress developing leaves were affected significantly less compared to developed leaves (Karim *et al.* 1999). However, genomic mechanisms behind differential tolerance between young and old leaves are not known.

Multiple studies have focused on understanding the adaptation of the model plant *Arabidopsis thaliana* to imposed heat stress (Quint *et al.* 2016; Dai Vu *et al.* 2019). In *Arabidopsis*, leaves emerging at different times vary in size, shape, growth rate and position and thus could be expected to vary in terms of photosynthetic capacity and stress tolerance. When we exposed 28-day-old *Arabidopsis* Col-0 plants to high-temperature stress under light at 42 °C, old leaves were damaged significantly more than young leaves based on visual observations. These observations were further confirmed by measurements of stress indicators such as tissue ion leakage, hydrogen peroxide (H_2_O_2_) and leaf necrosis. Although plants have been frequently studied when subjected to either moderate or severe heat stress treatments both in the context of basal thermotolerance and acquired thermotolerance, little is known about mechanisms behind young leaf’s tolerance to high-temperature stress compared to older leaf’s response.

Studies have shown that light and heat can produce an interactive effect, such that plants’ lack of phytochrome B (phyB) would increase tolerance to heat stress (Arico *et al.* 2019), and phyB has been verified to be essential as a thermal sensor in plants (Song *et al.* 2017). The activity of ascorbate peroxidase (APX) is induced by moderate heat stress and genes encoding APX such as APX1 and APX2 were dependent on heat shock factor (Hsf) (Panchuck *et al.* 2002). Auxin is a major coordinating signal in the regulation of heat stress. Multiple growth regulators including auxin, gibberellin (GA) and abscisic acid (ABA) have been identified to be involved in thermotolerance of plants. Auxin efflux carriers have a role in auxin transport from the shoot apical meristem (Petrášek and Friml 2009). When plants were exposed to several stresses, the reduction of GA levels had been observed, and increased GA biosynthesis promoted plant growth (Colebrook *et al.* 2014). Mutants impaired in ABA synthesis are sensitive to heat stress (Suzuki *et al.* 2016) and the multiprotein bridging factor 1c (MBF1c) overexpression lines show enhanced thermotolerance. Galactinol synthase (GolS), a key enzyme for the synthesis of the osmoprotectant during heat stress, has been implicated in thermotolerance and GolS was induced when HsfA2 was overexpressed (Nishizawa *et al.* 2008). Moreover, calcium transients have been suggested to have roles in heat stress signalling, as *Arabidopsis* lacking the transcription factor MYB30 protein had a greater elevation of [Ca^2+^]_cyt_ in response to heat and oxidative stress (Liao *et al.* 2017), and modification of [Ca^2+^]_cyt_ levels led to changes in thermotolerance in tobacco seedlings (Gong *et al.* 1998). More importantly, the elevated [Ca^2+^]_cyt_ can directly modulate the DNA-binding activity of Hsf, thus inducing expression of Hsp genes (Li *et al.* 2004). Thus, known plant adaptations to high-temperature stress are related to stress signalling, plant growth regulators, chaperone proteins and ROS homeostasis. To understand the mechanisms behind young leaf’s differential thermotolerance, we opted to use a transcriptomic approach to test whether any of these known mechanisms and potentially novel ones are involved.

RNA-seq experiments could generate transcriptome data which could be explored to identify gene expression patterns over time and space (Wan *et al.* 2011). Hence, we opted to use them to explore differences between the transcriptomes of young and old leaves. Our results indicated that younger leaves had significant differential expression of multiple genes under stress compared to the older leaves. Analyses of differentially expressed genes (DEGs) revealed that younger leaves highly expressed genes involved in known stress tolerance-related pathways such as heat shock proteins and in pathways less studied in connection to heat stress including pectin metabolism, cutin and wax biosynthesis, pentose and glucuronate interconversions, cellulose degradation, indole glucosinolate metabolism and RNA splicing.

## Materials and Methods

### Plant material and growing conditions

Seeds were purchased from Ohio State University Arabidopsis Biological Resource Center (ABRC). Wild-type *A. thaliana* Col-0 were imbibed in 0.1 % agar at 4 °C 3 days for vernalization. Plants were grown in propagation medium (Mix number 2, Farfard Inc., Agawam, MA, USA) under a constant condition at 22 °C with relative humidity ranging from 40 to 50 %. And lighting was provided at 4100-K fluorescent bulbs with a photoperiod of 16 h of 150 μmol m^−2^ s^−1^. For heat stress experiment, 28-day-old, container-grown plants were heat-stressed in a controlled environmental chamber with 90 % relative humidity under 42 °C started from 9:00 am. Three biological replicates of young and old leaves were collected for each of the three treatments (i) right before heat stress and (ii) after 1 h and (iii) 10 h heat stress. Immediately after collecting the samples, they were flash-frozen in liquid nitrogen, and stored at −80 °C until use for further analysis.

### Electrolyte leakage measurement

For determination of membrane injury index, young and old leaves from 28-day-old *Arabidopsis* plants were cut from stressed and control plants, stress conditions as described above. Samples with three replications of leaves were immersed in 10 mL of distilled water for 2 h at room temperature. After incubation, conductivity of the solutions was measured with a conductivity meter (YSI model 32 conductance meter). Samples were then boiled for 10 min and cooled to room temperature prior to measuring conductivity again. Injury index was determined using the formula:


$$\begin{array}{l} I\;(\%)=[1-(1-t_{1}/t_{2})/(1\;\;c_{1}/c_{2})]*100 \end{array}$$

And *t*_1_ and *t*_2_ are the first time and second (after boiling) time measurements of the conductivity of the water solutions from heat stress plants. And corresponding *c*_1_ and *c*_2_ are the first time and second time (after boiling) measurements of the conductivity of the water solutions from control plants (Premachandra *et al.* 1992).

### H_2_O_2_ measurement

Hydrogen peroxide content was quantified in leaves following the method described by Carvalho *et al.* and Junglee *et al* with modifications (Junglee *et al.* 2014; Carvalho *et al.* 2017). Young, intermediate and old leaf tissue (100 mg fresh weight) was ground in liquid nitrogen using 1 mL of 0.1 % (w/v) trichloroacetic acid (TCA). Homogenates of the samples were centrifuged at 12 000*g*, at 4 °C for 20 min, and 100 μL of the supernatant were added to a solution containing 250 μL of 10 mM potassium phosphate buffer (pH 7.0), 500 μL of 1 M potassium iodide and 150 μL of TCA 0.1 % (w/v). Then, the resulting solutions were kept on ice in the dark room for 30 min. Finally, absorbance at 350 nm was measured using a spectrophotometer. The resulting values are expressed as μmol H_2_O_2_·g FW^−1^ calculated via comparison to a standard curve.

### RNA extraction and next generation of RNA-sequencing library

RNA-sequencing (RNA-seq) work plan was designed to compare transcript abundances between young leaves and old leaves when the whole plants were exposed to normal (23 °C) or 1 h or 10 h at high-temperature stress (42 °C) conditions. Total RNA was isolated from leaves using the Plant RNeasy mini kit (Qiagen), followed by on-column DNase treatment. RNA quality and concentrations were determined by both UV spectrophotometry and Agilent Bioanalyser. RNA-seq libraries were constructed for 18 RNA samples by following instructions for RNA library prep kit for Illumina (NEBNext Ultra protocol #E7530S/L). Barcoded cDNA libraries were constructed from 500 ng of total mRNA with three biological replicates for each of the three treatments. The first-strand cDNA synthesis incubation was set at 42 °C for 30 min in the preheated thermal cycler. The 18 samples were diluted to 5 nM based upon their template length and template concentration. And checks on library quality on a Bioanalyser showed a narrow distribution with a peak size approximately 300 bp. Sequencing was performed using an Illumina NextSeq 500 instrument with 2 × 150 cycles MID output (generating 150-bp paired-end reads) at the University of Florida Interdisciplinary Center for Biotechnology Research. Replicates were run in four separate lanes, with a total of 18 samples from different tissues and different treatments in each lane. The sequencing reads were deposited in NCBI’s Gene Expression Omnibus and are accessible to download through GEO Series accession number (SRA submission no. SUB6541848).

### Quantification of transcripts

Residual adaptor sequences were removed from raw reads and ‘paired output’ was selected to keep the two paired read files synchronized avoiding any unpaired reads resulting from trimming. Each RNA-seq read was trimmed using Trimmomatic (Bolger *et al.* 2014) to make the average quality score threshold set at 20 and the minimum length 20 bp. Each sample’s sequence file from four lanes was concatenated. The sequencing files were examined after concatenation using FASTQ Groomer version 0.36.5. All trimmed reads were mapped to the *A. thaliana* reference transcript data set AtRTD2-QUASI containing 82 190 non-redundant transcripts (Brown *et al.* 2017) using the alignment tool HISAT 2 (Kim *et al.* 2015), allowing up to two base mismatches per read. Reads mapped to multiple locations were discarded. Given the SAM/BAM file and the reference transcript, we count for each gene of how many aligned reads overlap its exons through HTSeq count tool (Anders *et al.* 2015). After obtaining count per million data sets, we divided read counts by the length of each gene in kilobases to derive ‘reads per kilobase’ (RPK), and then RPK in a sample was divided by 1 million to get transcripts per million (TPM). Using transcript abundance data and the differential expression of isoforms was tested based on the negative binomial model through DESeq2 package in R (Di *et al.* 2011). The thresholds for DEGs were filtered by the threshold of |fold change| > 2 (log_2_ scale) and adjusted *P*-values < 0.01 for the null hypothesis. Results of expression of transcripts are shown in **Supporting Information—Tables S1****–****S9**.

### Identification of DEGs

The set of heat stress-responsive genes differentially expressed in young leaves was determined by performing several pairwise DEseq comparisons of young leaf to old leaf: (i) Col-0 young leaf grown under normal condition to Col-0 old leaf grown under normal conditions, (ii) Col-0 young leaf from plants exposed to 1 h heat stress condition to Col-0 old leaf from plants exposed to 1 h heat stress, (iii) Col-0 young leaf from plants exposed to 10 h heat stress to Col-0 old leaf from plants exposed to 10 h heat stress. Transcript per million data sets of DEGs that were upregulated or downregulated in young leaf relative to old leaf were compiled for 0 h, 1 h and 10 h heat stress conditions. The first comparison of young and old leaves at the non-heat condition (right before heat stress), and the detection of differential gene isoforms were based on different developmental stages only. And this information as a reference was used further to compare with heat stress-induced changes in transcript isoforms. In the second comparison of transcripts in young and old leaves at 1 h heat stress, young leaves showed upregulated expression of transcripts for multiple genes annotated as Hsp and Hsf compared with old leaves. Genes that were differentially expressed in young and old leaf were retained in four sets (upregulated in young leaf, downregulated in young leaf, upregulated in old leaf, downregulated in old leaf). A Venn diagram of these sets was used to compile a list of 1660 upregulated genes and 910 downregulated genes. These were subsequently filtered by removing genes that were less than 5-fold (|log_2_FC| > 2) higher or lower in young leaf to old leaf. This list was then compared to DEGs that responded to 0 h, 1 h, 10 h heat stress between young leaf and old leaf, and genes that have lower fold changes to one of these treatments were removed. To extract co-expression networks, gene ID and fold change values were analysed using the STRING database to identify the functional clusters, their enrichment scores and false discovery rates (Szklarczyk *et al.* 2021).

## Results

### Old leaves were damaged significantly more than young leaves under heat stress

**Figure 1.**
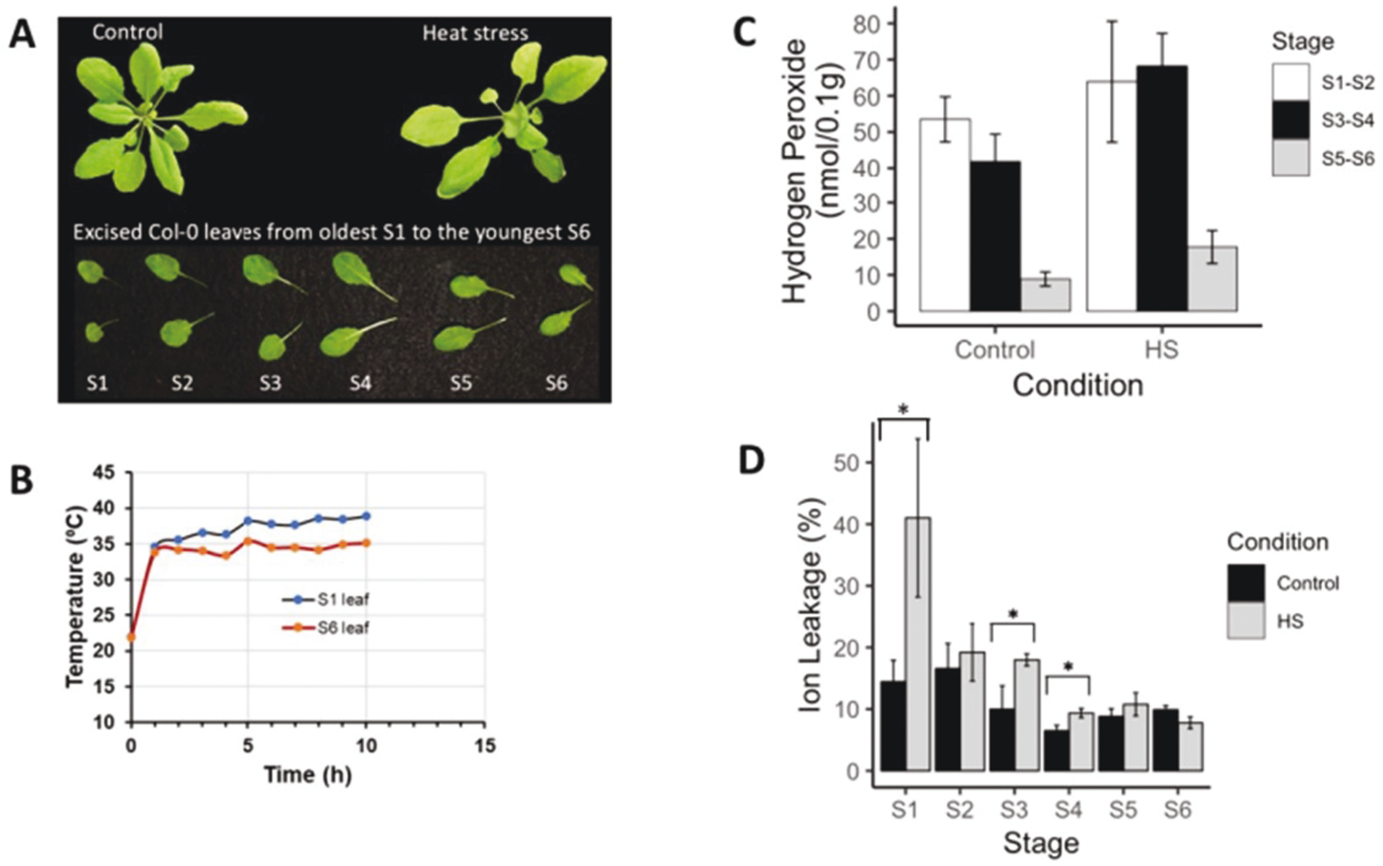
*Arabidopsis thaliana* wild-type Col-0 young and old leaves comparisons under control and 42° heat stress (HS) treatment. (A) Phenotypes of 28-day-old Col-0 under normal growth environment and under 42° HS for 10 h. (B) Leaf temperature over time for S1 and S6 leaves recorded using an infrared thermometer on plants placed at HS treatment. Mean (*n* = 3) values are shown and the standard errors were less than 2.3 % of the mean. (C) Leaf hydrogen peroxide content under control and HS conditions. (D) Ion leakage percentages of different stages under control and HS treatment. Difference between mean ion leakage percentage values in stage 1, 3 and 4 was significantly different at *P* < 0.05 (*), as determined by Student’s *t*-test.

To better define the heat stress damage to rosette leaf tissues, we subjected whole plants to high-temperature stress and analysed young and old leaves separately. We used *Arabidopsis* plants at growth stage 1.12 with 12 rosette leaves each above 1 mm in length, and different stages of leaves were distinguishable (Boyes *et al.* 2001). First, we assessed the difference between young and old leaves immediately following exposure of plants to 42 °C heat treatment. Following incubation of plants in the high temperature for 10 h, the young leaves had movement with the elevation angle close to 90° (Fig. 1A). When surface temperatures were measured, younger leaf (S6) and older leaf (S1) did not differ significantly up to two hours but S1 leaf registered a significantly higher temperature at subsequent times (Fig. 1B). Both of the older stage leaves S1–S2 and S3–S4 have similar level of ROS accumulation in terms of H_2_O_2_ measurement. Hydrogen peroxide accumulation was about three times higher in the older stage leaves (S1–S2) than the younger stage leaves (S5–S6) both under control and heat stress conditions (Fig. 1C). Oldest leaf S1 was the most vulnerable among all the leaf stages since heat stress induced about double the ion leakage percentage compared with the control condition, while both S5 and S6 had the least effects from heat damage (Fig. 1D).

### Transcriptomic overview

**Figure 2.**
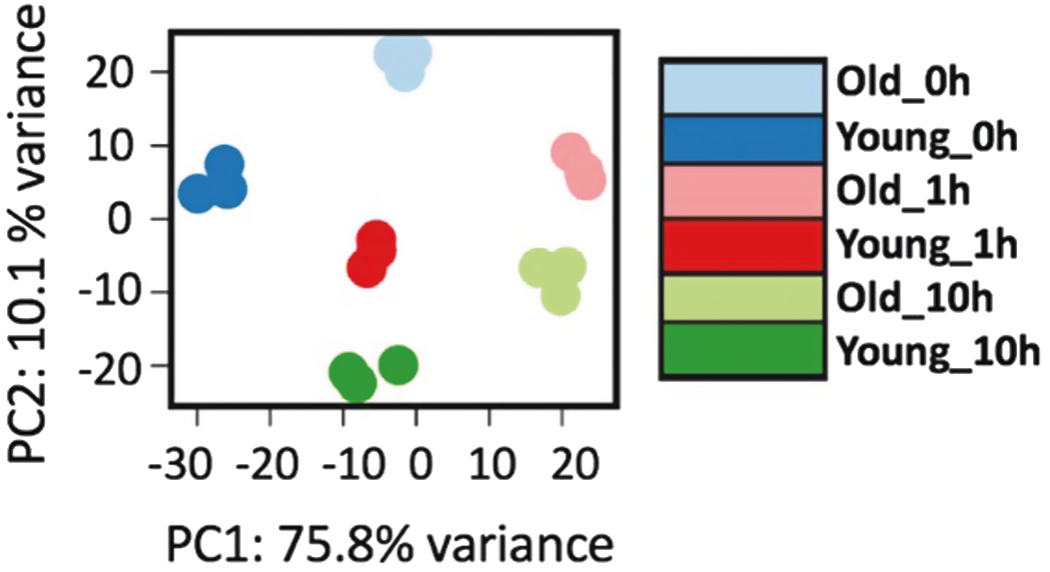
Differentially expressed genes between young and old leaves under control, 1 h and 10 h heat stress conditions. Principal component analysis (PCA) of 18 samples from the RNA-seq experiment.

The wild-type Col-0 28-day-old plants were subjected to 42 °C for 0 h, 1 h and 10 h before collecting S1 and S6 tissue separately. The numbers of DEGs were achieved by comparing transcripts in young leaf S6 relative to old leaf S1. Initial analyses of differential groups of comparisons (Groups A–I) allowed us to collect all the DEG using the threshold |log_2_FC| of over 2 **[see****Supporting Information—Tables S1****–**S**9****]**. Groups A–C were derived by dividing transcript abundance in young leaf by corresponding transcript abundance in the old leaf under control, 1 h and 10 h heat stress condition, respectively **[see****Supporting Information—Tables S1****–****S3****]**. Groups D–I were for comparing heat-stressed samples with corresponding control samples **[see****Supporting Information—Tables S4****–****S9****]**, The TPM values of the 18 different RNA-seq samples were analysed by principal component analysis (PCA, Fig. 2). The first two components (PCs) explained 75.8 % and 10.1 % of the total variance, respectively. Based upon the dot separation of the 18 samples of six conditions with three replications, the variations among the three replications are smaller than the variation among the heat stress treatments. The first PC can separate Young_0h from Old_1h and Old_10h and the second PC can separate Old_0h from Young_10h. Both PCs contributed to the separation of tissue type and stress conditions.

**Figure 3.**
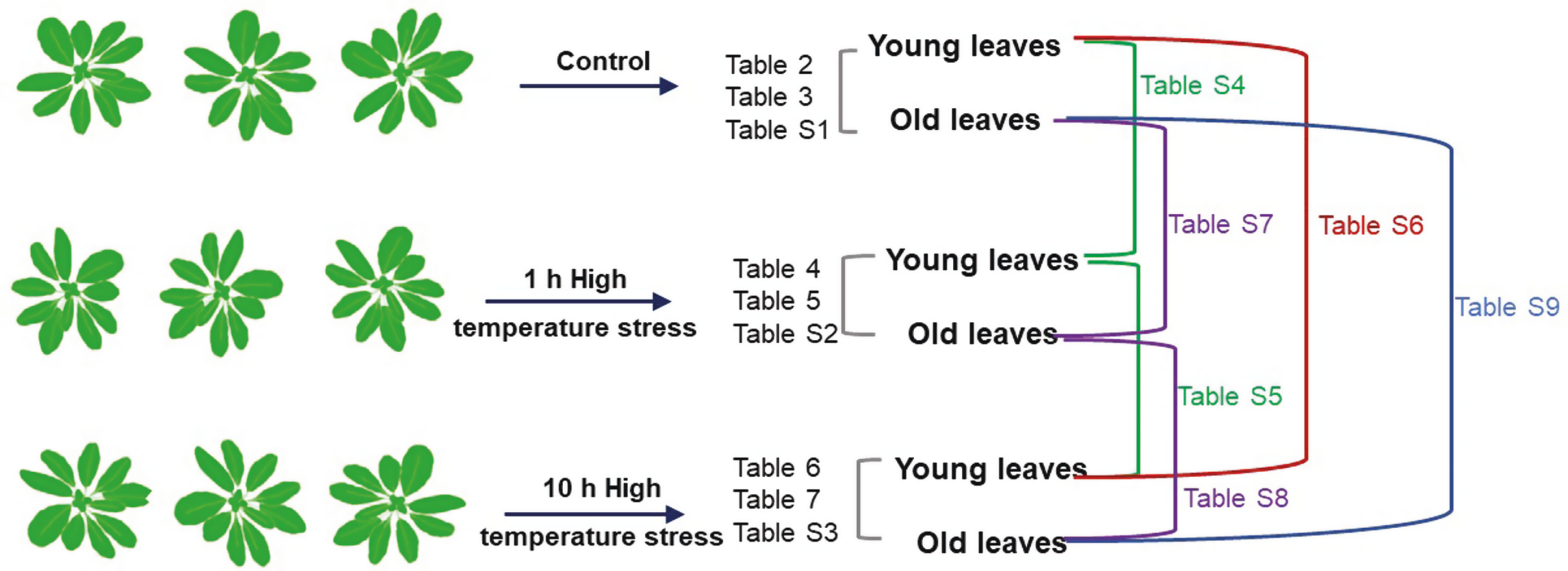
Comparisons of gene expression levels in leaf tissue samples made in this study indicating specific tables where data summaries are presented.

Gene expression levels were compared between different treatments to determine significant fold changes and are presented in a series of tables as indicated in Fig. 3. To examine the distribution of the DEG with higher or lower levels of transcripts in young leaf compared with old leaf, we extracted the DEG lists under different conditions. For this analysis we considered only those transcripts that have a base mean (the mean value of transcript counts for all the three conditions) of 10 or more, and the fold change between young and old leaves to be significant at the adjusted *P*-value ≤ 0.05 (Table 1). A total of 3395, 4486 and 3651 transcripts were expressed at greater level (log_2_FC 0.2 to 7.9) in young leaves than the old leaves under control, 1 h and 10 h stress, respectively (Table 1). A total of 3077, 4640 and 3240 transcripts were expressed at lower level (log_2_FC −0.8 to −8.2) in young leaves than the old leaves under control, 1 h and 10 h stress, respectively (Table 1). Data mining of these DEGs using STRING database (Szklarczyk *et al.* 2021) revealed between 4 and 95 functional clusters of genes (Table 1).

**Table 1.** Differentially expressed genes based on significant difference between young and old leaves of *Arabidopsis thaliana* (adjusted *P* ≤ 0.05) under control and two levels of heat stress conditions. The numbers of genes that were greater or lower in transcript abundance in the young leaves based on fold change (log_2_FC) compared to the old leaves were identified from analyses using DESeq2 and the functional clusters of genes were identified by data mining using STRING (version 11.5).

Treatment	Log_2_(FC) for the comparison young versus old leaves	Number of genes	Number of functional clusters
Control	0.4 to 8.5	3395	53
Control	−0.4 to −7.6	3077	4
Heat stress, 1 h	0.2 to 8.6	4486	27
Heat stress, 1 h	−0.2 to −4.9	4640	22
Heat stress, 10 h	0.3 to 7.9	3651	95
Heat stress, 10 h	−0.3 to −8.2	3240	14

### DEGs under control conditions

Under control conditions, genes known in photosynthesis, spliceosome Saf4/Yju2, protein import, proteasome, stomatal complex, cell division, pectin metabolism and ribosomal proteins were expressed significantly greater in young leaves than old leaves (Table 2). In contrast, the genes that were expressed significantly lower in young leaves compared to old leaves were those related to defence and ageing (Table 3).

**Table 2.** Descriptions of functional clusters of genes identified by data mining of genes expressed at significantly greater levels in young leaves than old leaves under control conditions, their enrichment scores, the number of genes mapped and the false discovery rate from an analysis using STRING (version 11.5). Descriptions which had adjectives ‘mixed’ or ‘mostly uncharacterized’ are marked with an asterisk (*).

Description	Enrichment score	Genes mapped	False discovery rate
Proteasome accessory complex	8.73	3	0.0041000
Photosystem I psaG/psaK, and photosystem I reaction centre subunit XI	8.39	4	0.0023000
Cellulose biosynthesis, and microtubule minus-end (*)	8.21	4	0.0042000
Photosystem I reaction centre, and photosystem I psaG/ psaK	8.12	5	0.0009900
Proteasome	8.03	4	0.0082000
TMEM14 family, and intracellular lipid transport (*)	7.55	5	0.0092000
COPI vesicle coat, and ER lumen protein-retaining receptor (*)	7.21	10	0.0001600
Protein export, and oligosaccharyltransferase complex	6.85	8	0.0045000
Photosystem I, and photosystem II stabilization	6.61	17	0.0000021
Proton-transporting two-sector ATPase complex	6.25	11	0.0029000
COPI vesicle coat, and Sec7 domain (*)	6.23	11	0.0032000
Respiratory chain, and NADH dehydrogenase (quinone) activity	6.09	13	0.0009300
ER–Golgi transport, and ArfGAP domain superfamily (*)	5.95	15	0.0008900
Protein export, and response to endoplasmic reticulum stress	5.86	13	0.0029000
Thylakoid membrane organization, and protein export (*)	5.38	14	0.0092000
Protein import into nucleus, and nuclear pore	5.37	15	0.0055000
Spliceosome, and Saf4/Yju2 protein	5.23	19	0.0020000
Aminoacyl-tRNA biosynthesis	5.19	17	0.0055000
NAD(P)H dehydrogenase complex (plastoquinone), and plastid thylakoid lumen (*)	4.95	26	0.0092000
Photosystem II assembly, and plastid thylakoid lumen (*)	4.95	18	0.0090000
Initiation factor, and programmed cell death protein 4	4.88	24	0.0042000
Plastid thylakoid lumen, and NAD(P)H dehydrogenase complex (plastoquinone)	4.52	53	0.0000720
photosystem, and thylakoid	4.49	94	0.0000001
Photosystem I, and photosynthesis	4.48	36	0.0001400
Extracellular matrix, and 3-oxo-cerotoyl-CoA synthase activity (*)	4.30	6	0.0000287
Pollen exine formation, and anther wall tapetum development (*)	4.05	3	0.0100000
Glutaredoxin-like, and response to lithium ion (*)	3.92	6	0.0001400
Glutaredoxin-like, and cytochrome c oxidase subunit 5c (*)	3.22	8	0.0001500
Cuticle development, and Bet v I type allergen (*)	2.84	15	0.0000021
Stomatal complex morphogenesis, and response to low humidity	2.64	7	0.0040000
Early nodulin-like protein domain, and Dullard phosphatase domain, eukaryotic (*)	2.18	16	0.0000526
Stomatal complex morphogenesis, and regulation of stomatal complex development	2.13	9	0.0055000
Cutin, suberine and wax biosynthesis, and fatty acid elongation (*)	1.91	50	0.0000001
Phragmoplast microtubule organization, and embryo sac cellularization (*)	1.90	12	0.0029000
DNA replication initiation, and DNA replication factor CDT1 like	1.82	11	0.0060000
Syntaxin, and TPX2, C-terminal (*)	1.72	47	0.0000000
DNA replication initiation, and GINS complex protein (*)	1.59	15	0.0042000
Mitosis, and ATP-dependent microtubule motor activity, plus-end-directed	1.34	35	0.0000012
Meiosis II, and microtubule-associated complex (*)	1.33	65	0.0000000
kinesin complex, and mitosis	1.20	39	0.0000099
DNA condensation, and condensed chromosome, centromeric region (*)	1.15	21	0.0079000
Cell division, and microtubule binding	1.13	64	0.0000000
DNA replication, and DNA polymerase III complex	1.09	34	0.0000188
Cell division, and microtubule-based movement	1.08	67	0.0000000
Mitotic cell cycle phase transition, and anaphase-promoting complex	1.02	25	0.0055000
Proteoglycan, and multicopper oxidase (*)	0.98	45	0.0000043
DNA replication, and DNA damage (*)	0.90	59	0.0000000
plant-type secondary cell wall biogenesis, and regulation of secondary cell wall biogenesis (*)	0.84	24	0.0007400
Pectin lyase fold, and pectin acetylesterase (*)	0.82	24	0.0009400
Homologous recombination, and mismatch repair (*)	0.67	21	0.0035000
cytosolic large ribosomal subunit, and ribosomal protein P1/P2, N-terminal domain	0.21	92	0.0000169
cytosolic large ribosomal subunit, and ribosomal protein S13/S18	0.14	72	0.0001100

**Table 3.** Descriptions of functional clusters of genes identified by data mining of genes expressed at lower levels in young leaves than old leaves under control conditions, their enrichment scores, the number of genes mapped and the false discovery rate from an analysis using STRING (version 11.5). Descriptions which had adjectives ‘mixed’ or ‘mostly uncharacterized’ are marked with an asterisk (*).

Description	Enrichment score	Genes mapped	False discovery rate
Berberine/berberine-like, and ageing (*)	1.45	32	0.0000013
Ageing, and methyltransferase type 11 (*)	1.59	24	0.0001500
Systemic acquired resistance, and cellular response to salicylic acid stimulus (*)	1.36	32	0.0025000
Gnk2-homologous domain, and positive regulation of defence response to oomycetes (*)	1.43	22	0.0066000

Upon 1 h heat stress, young leaves had significantly increased transcripts for genes related to proteasome complex, ribosome biogenesis, mRNA surveillance, cutin, suberin and wax synthesis, stomatal development, secondary cell wall biogenesis, pentose and glucuronate interconversions and pectin metabolism (Table 4). In contrast, the genes that were expressed at lower levels in young leaves included genes related to plastid thylakoid lumen and NADPH dehydrogenase complex, RNA polymerase, photosystem, hypoxia, ageing- and defence-related gene networks (Table 5).

**Table 4.** Descriptions of functional clusters of genes identified by data mining of genes expressed at greater levels in young leaves than old leaves after 1 h of heat stress, their enrichment scores, the number of genes mapped and the false discovery rate from an analysis using STRING (version 11.5). Descriptions which had adjectives ‘mixed’ or ‘mostly uncharacterized’ are marked with an asterisk (*).

Description	Enrichment score	Genes mapped	False discovery rate
SNARE-associated Golgi protein, and serine incorporator (Serinc) (*)	8.87	3	0.0078000
Proton-transporting V-type ATPase complex	7.77	7	0.0026000
RNA degradation, and Ataxin 2 SM domain (*)	6.59	13	0.0033000
Proteasome complex	6.54	11	0.0096000
Preribosome, large subunit precursor, and Brix domain (*)	6.44	15	0.0021000
Extracellular matrix, and 3-oxo-cerotoyl-CoA synthase activity (*)	6.23	6	0.0000119
mRNA surveillance pathway, and mRNA *cis*-splicing, via spliceosome (*)	6.00	15	0.0078000
Ribosome biogenesis, and DEAD/DEAH box helicase domain (*)	5.73	24	0.0034000
Initiation factor, and programmed cell death protein 4	5.49	19	0.0097000
Glutaredoxin-like, and response to lithium ion (*)	5.22	5	0.0009400
Ribosome biogenesis in eukaryotes, and ribosome biogenesis (*)	4.96	61	0.0000349
Response to lithium ion, and putative lipid-transfer protein DIR1-like (*)	4.89	4	0.0070000
ER–Golgi transport, and ArfGAP domain superfamily (*)	4.57	34	0.0033000
Cuticle development, and Bet v I type allergen (*)	3.29	17	0.0000119
Ribosomal subunit (*)	3.17	160	0.0002300
Ascorbate oxidase homologue, first cupredoxin domain and guard cell development	3.08	9	0.0054000
Cutin, suberine and wax biosynthesis, and fatty acid elongation (*)	2.85	48	0.0000022
Stomatal complex morphogenesis, and regulation of stomatal complex development	2.69	10	0.0088000
Plant-type secondary cell wall biogenesis, and regulation of secondary cell wall biogenesis (*)	2.21	22	0.0067000
Multicopper oxidase, and phosphoribosyltransferase C-terminal (*)	2.21	22	0.0018000
Proteoglycan, and multicopper oxidase (*)	1.98	51	0.0000020
Pentose and glucuronate interconversions, and galactokinase, N-terminal domain (*)	1.90	19	0.0039000
Pectin lyase fold, and pectin acetylesterase (*)	1.71	38	0.0069000
Leucine-rich repeat-containing N-terminal, plant-type, and malectin-like domain	1.07	41	0.0069000
rRNA binding, and ribosomal protein L35, conserved site (*)	0.47	26	0.0067000
Plastid ribosome, and rRNA binding (*)	0.43	30	0.0034000
rRNA binding, and organellar large ribosomal subunit (*)	0.29	35	0.0035000

**Table 5.** Descriptions of functional clusters of genes identified by data mining of genes expressed at lower levels in young leaves than old leaves after 1 h of heat stress, their enrichment scores, the number of genes mapped and the false discovery rate from an analysis using STRING (version 11.5). Descriptions which had adjectives ‘mixed’ or ‘mostly uncharacterized’ are marked with an asterisk (*).

Description	Enrichment score	Genes mapped	False discovery rate
Nucleosome core, and centromere kinetochore component CENP-T histone fold	5.87	7	0.0032000
NAD(P)H dehydrogenase complex (plastoquinone), and plastid thylakoid lumen (*)	5.65	9	0.0016000
Plastid thylakoid lumen, and NAD(P)H dehydrogenase complex (plastoquinone) (*)	5.59	11	0.0002700
ER body, and coumarin metabolic process (*)	5.36	5	0.0002400
G protein-coupled receptor signalling pathway, and haemolysin-III related (*)	4.90	12	0.0065000
Thylakoid, and stromule	4.30	20	0.0021000
Nuclear DNA-directed RNA polymerase complex, and basal transcription factors (*)	4.23	27	0.0049000
ER body, and Jacalin-like lectin domain, plant (*)	4.22	7	0.0004000
Phosphatase complex, and calcineurin-like phosphoesterase (*)	4.20	18	0.0065000
Photosystem, and thylakoid	4.19	18	0.0065000
Nuclear DNA-directed RNA polymerase complex, and basal transcription factors (*)	4.09	31	0.0093000
Multivesicular body sorting pathway, and regulator of Vps4 activity in the MVB pathway (*)	3.50	33	0.0095000
Gnk2-homologous domain, and positive regulation of defence response to oomycetes (*)	2.31	16	0.0015000
Cyanoamino acid metabolism, and cellulose degradation (*)	1.88	19	0.0051000
CF(0), and reverse transcriptase, RNA-dependent DNA polymerase (*)	1.75	20	0.0085000
Cytochrome c-type biogenesis, and proton-transporting ATP synthase complex, coupling factor F(o) (*)	1.64	34	0.0000001
Berberine/berberine-like, and ageing (*)	1.61	23	0.0013000
Cytochrome c-type biogenesis, and mechanosensitive ion channel MscS domain superfamily (*)	1.16	45	0.0002400
Calmodulin-binding protein-like, and wall-associated receptor kinase C-terminal (*)	1.08	27	0.0016000
Indole glucosinolate metabolic process, and regulation of salicylic acid biosynthetic process (*)	0.91	36	0.0016000
Indole glucosinolate metabolic process, and defence response to bacterium, incompatible interaction (*)	0.87	44	0.0000689
Response to chitin, and cellular response to hypoxia (*)	0.63	84	0.0001600

Upon 10 h of heat stress, the genes with significantly higher transcript abundance in young leaves were those related to ribosome synthesis and function, aminoacyl-tRNA synthesis, spliceosome, respirasome, porphyrin synthesis, protein folding and DNAJ domain proteins, stress-related transcription factors, pectin acetylesterase and beta-glucanase, pectin lyase, synthesis of cutin, suberin and wax synthesis, cell division, secondary wall synthesis and DNA replication (Table 6). The functional clusters of genes that were significantly less abundant in young leaves at this stress level were those involved in mRNA splicing, proteasome ubiquitin homologues, cellulose synthase and defence-related genes (Table 7).

**Table 6.** Descriptions of functional clusters of genes identified by data mining of genes expressed at greater levels in young leaves than old leaves after 10 h of heat stress, their enrichment scores, the number of genes mapped and the false discovery rate from an analysis using STRING (version 11.5). Descriptions which had adjectives ‘mixed’ or ‘mostly uncharacterized’ are marked with an asterisk (*).

Description	Enrichment score	Genes mapped	False discovery rate
Proton-transporting V-type ATPase complex	8.90	3	0.0050000
Prp19 complex, and U5 snRNA binding (*)	8.74	3	0.0089000
Preribosome, large subunit precursor, and Brix domain (*)	8.02	8	0.0002500
Respiratory chain complex I	7.74	7	0.0028000
Chloroplast nucleoid, and Group II intron splicing (*)	7.70	6	0.0065000
Ribosome biogenesis in eukaryotes, and ribosome biogenesis (*)	7.68	30	0.0000000
Aminoacyl-tRNA biosynthesis, and amidase (*)	7.49	13	0.0000387
Plastid translation, and CCB3/YggT (*)	7.47	14	0.0000086
Respiratory chain complex I	7.42	9	0.0020000
Ribosome biogenesis in eukaryotes, and protein of unknown function DUF1068	7.39	11	0.0003400
Initiation factor	7.37	11	0.0003400
Plastid translation, and small ribosomal subunit rRNA binding	7.37	9	0.0026000
Proton-transporting two-sector ATPase complex, and inorganic pyrophosphatase	7.36	13	0.0000820
Aminoacyl-tRNA biosynthesis	7.36	11	0.0003400
Ribosome biogenesis in eukaryotes, and preribosome, small subunit precursor	7.31	9	0.0032000
ATP synthesis, and ATP synthase, F0 complex, subunit b	7.22	8	0.0068000
Spliceosome, and Saf4/Yju2 protein	7.22	13	0.0001000
Translation preinitiation complex, and initiation factor	7.16	8	0.0082000
GroEL-like equatorial domain superfamily, and GroES chaperonin family	6.92	17	0.0000563
Protein export, and oligosaccharyltransferase complex	6.78	12	0.0026000
Respirasome, and plastoquinone	6.66	29	0.0000121
Protein export, and response to endoplasmic reticulum stress	6.64	14	0.0016000
Plastid ribosome, and rRNA binding	6.55	12	0.0054000
Cytosolic large ribosomal subunit	6.35	16	0.0017000
Porphyrin biosynthesis, and magnesium chelatase complex	6.32	13	0.0061000
Respiratory chain, and NADH dehydrogenase (quinone) activity	6.24	21	0.0020000
Cytosolic large ribosomal subunit	6.16	23	0.0005900
COPI vesicle coat, and ER lumen protein-retaining receptor	6.15	18	0.0015000
Mitochondrial ribosome, and ribosomal protein	6.06	15	0.0050000
Mixed, incl. extracellular matrix, and 3-oxo-cerotoyl-CoA synthase activity	6.05	6	0.0000037
ER–Golgi transport, and ArfGAP domain superfamily (*)	5.95	28	0.0035000
Cytosolic large ribosomal subunit, and ribosomal protein S13/S18 (*)	5.89	66	0.0000000
Protein folding, and Hsp90 protein binding (*)	5.84	36	0.0000387
Cytosolic large ribosomal subunit, and ribosomal protein S13/S18	5.74	43	0.0000240
Protein folding, and DNAJ domain	5.63	54	0.0000019
Cytosolic large ribosomal subunit, and ribosomal protein P1/P2, N-terminal domain	5.57	84	0.0000000
Glutaredoxin-like, and response to lithium ion (*)	5.50	6	0.0000278
Ribosomal subunit	5.43	150	0.0000000
Microtubule sliding, and EB1, C-terminal	5.32	5	0.0003400
Cytosolic small ribosomal subunit	5.25	66	0.0000002
Ribosomal protein, and mitochondrial translational elongation	5.17	45	0.0010000
Ribosomal protein	5.05	43	0.0020000
Glutaredoxin-like, and cytochrome c oxidase subunit 5c (*)	4.99	8	0.0000114
Response to lithium ion, and putative lipid-transfer protein DIR1-like	4.97	5	0.0010000
bZIP Maf transcription factor, and water stress and hypersensitive response (*)	4.62	4	0.0065000
Early nodulin-like protein domain, and Dullard phosphatase domain, eukaryotic (*)	4.60	16	0.0000000
Associate of Myc 1, and putative cell wall protein (*)	4.23	5	0.0068000
Pectin acetylesterase, and beta-glucanase (*)	3.44	7	0.0082000
Syntaxin, and TPX2, C-terminal (*)	3.21	55	0.0000000
Phragmoplast microtubule organization, and embryo sac cellularization (*)	2.95	12	0.0013000
TFIIS/LEDGF domain superfamily, and response to UV-A (*)	2.68	13	0.0022000
Meiosis II, and microtubule-associated complex (*)	2.48	76	0.0000000
Cutin, suberine and wax biosynthesis, and fatty acid elongation (*)	2.34	63	0.0000015
Cyclin, and cyclin-dependent protein kinase activity (*)	2.28	16	0.0054000
Mitosis, and ATP-dependent microtubule motor activity, plus-end-directed (*)	2.17	31	0.0000374
Kinesin complex, and mitosis (*)	2.09	33	0.0000563
Cell division, and microtubule binding (*)	1.96	55	0.0000020
Proteoglycan, and multicopper oxidase (*)	1.88	55	0.0000000
Cell division, and microtubule-based movement	1.80	58	0.0000157
Multicopper oxidase, and phosphoribosyltransferase C-terminal (*)	1.75	22	0.0055000
Pectin lyase fold, and pectin acetylesterase (*)	1.67	40	0.0023000
Plant-type secondary cell wall biogenesis, and regulation of secondary cell wall biogenesis (*)	1.26	29	0.0002300
Xylan metabolic process, and regulation of secondary cell wall biogenesis (*)	1.00	35	0.0003500
DNA replication, and DNA damage (*)	0.92	41	0.0012000

**Table 7.** Descriptions of functional clusters of genes identified by data mining of genes expressed at lower levels in young leaves than old leaves after 10 h of heat stress, their enrichment scores, the number of genes mapped and the false discovery rate from an analysis using STRING (version 11.5). Descriptions which had adjectives ‘mixed’ or ‘mostly uncharacterized’ are marked with an asterisk.

Description	Enrichment score	Genes mapped	False discovery rate
mRNA *cis*-splicing, via spliceosome, and U2AF complex	6.61	3	0.0064000
Chloroplast nucleoid, and Group II intron splicing (*)	5.22	11	0.0030000
mRNA surveillance pathway, and mRNA *cis*-splicing, via spliceosome (*)	4.88	15	0.0026000
Chloroplast nucleoid, and Group II intron splicing (*)	4.84	16	0.0026000
Proteasome, and ubiquitin homologues (*)	4.81	14	0.0044000
Gnk2-homologous domain, and positive regulation of defence response to oomycetes	2.74	13	0.0004700
Positive regulation of defence response to oomycetes, and proline-rich membrane anchor 1	2.72	8	0.0046000
Aluminium cation transport, and atrichoblast differentiation	1.94	15	0.0032000
Berberine/berberine-like, and ageing (*)	1.80	24	0.0026000
Systemic acquired resistance, and cellular response to salicylic acid stimulus (*)	1.79	22	0.0032000
Zinc finger, and cellulose synthase (*)	1.09	26	0.0097000
Indole glucosinolate metabolic process, and defence response to bacterium, incompatible interaction (*)	0.74	30	0.0026000
Response to chitin, and cellular response to hypoxia	0.47	52	0.0068000

### Heat map of DEGs

**Figure 4.**
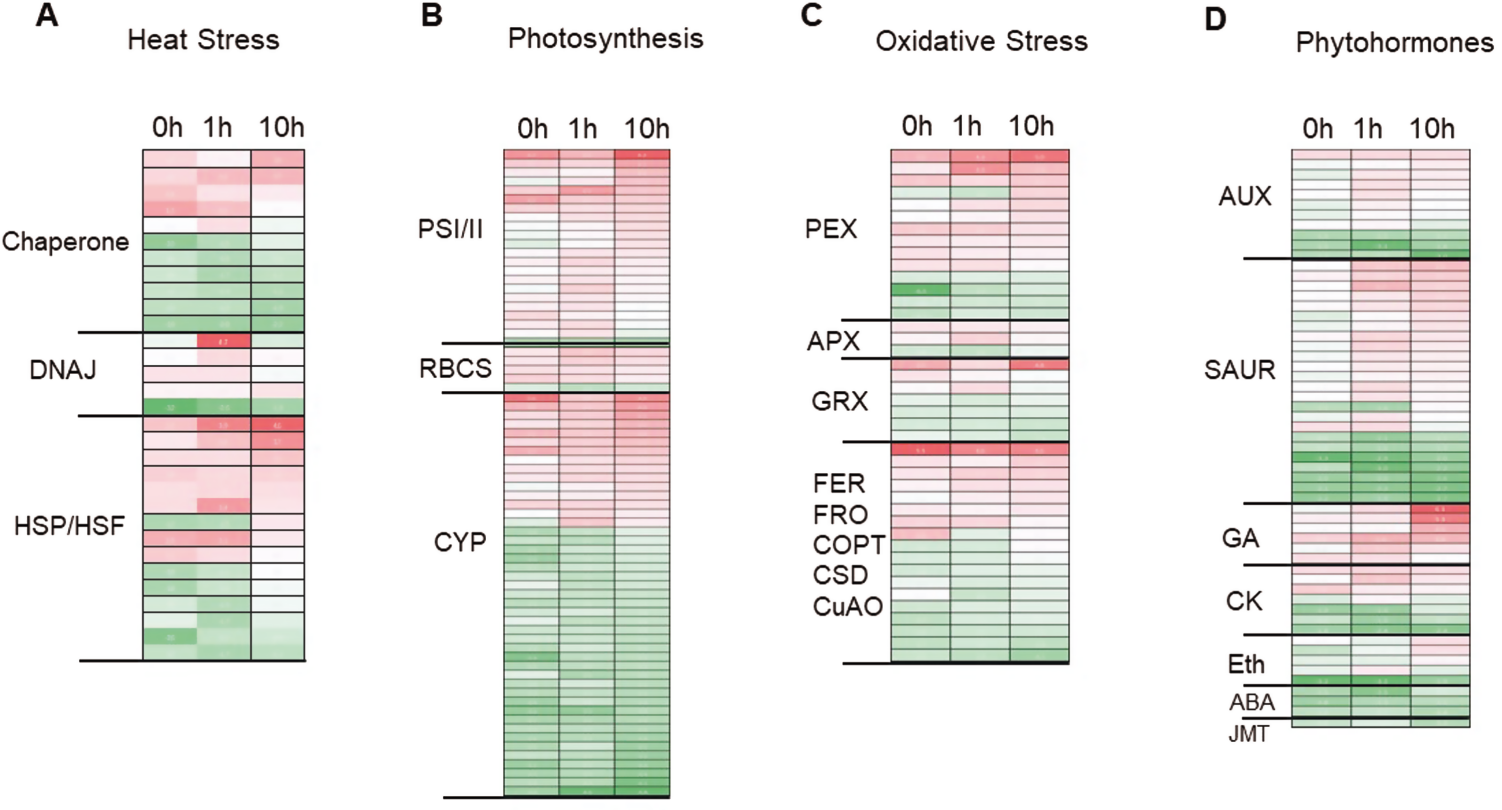
Heatmap of grouping differentially expressed genes between young and old leaf. (A). Differentially expressed genes involved in chaperone, DNAJ heat shock protein and HSP/HSF. (B) Differentially expressed genes involved in photosynthesis and photorespiration including genes involved in PSI and PSII electron transport, RUBISCO small subunit protein family (RBCS) and cytochrome P450 (CYP). (C) Differentially expressed genes involved in oxidative stress including peroxidase (PEX), ascorbate peroxidase (APX), glutaredoxin (GRX), ferritin (FER), ferric reduction oxidase (FRO), copper transport proteins (COPT), copper amine oxidase (CuAO) and copper superoxide dismutase (CSD). (D) Differentially expressed genes involved in phytohormones like auxin (AUX), gibberellin (GA), cytokinin (CK), ethylene (Eth), abscisic acid (ABA), jasmonic acid (JMT). Red colour indicates genes whose transcripts are significantly higher in young leaf compared with old leaf and green colour indicate genes whose transcripts are significantly lower in young leaf compared with old leaf. For colour, please refer to the online images.

To further explore the differential impacts of high temperature on young leaf and old leaf, we grouped gene families that were previously reported to be related to heat stress tolerance (Larkindale and Vierling 2008). In particular, we focused on genes encoding proteins that were involved in heat stress signalling such as chaperones, DNAJ heat shock proteins, Hsp, Hsf and regulation of photosystem I/II subunit, cytochrome P450 and oxidative stress-related proteins peroxidase, APX, glutaredoxin, ferredoxin, copper/iron superoxide dismutase; genes involved in phytohormone biosynthesis such as auxin, GA, cytokinin, ethylene, ABA, jasmonic acid, and cell wall expansion and biosynthesis genes encoding xyloglucan hydrolase, expansin, pectin lyase-like superfamily protein, pectin methylesterase inhibitor superfamily protein, pectin acetylesterase family protein, pectin methylesterase and cellulose synthase-like protein (Fig. 4; **see****Supporting Information—Tables S10****–****S14**). In Fig. 4, the bright red colour in heatmap indicates higher DEG in young leaf compared with old leaf, and the dark green colour indicates higher DEG in old leaf compared with young leaf. Hsf genes, Hsf A4A, Hsf A8 and Hsf B2A, were downregulated in young leaf compared with old leaf after 1 h heat stress (Fig. 4; **see****Supporting Information—Table S10**). Both Hsp 70 and Hsp 90 greatly increased TPM under heat stress, while young leaf had two times higher TPM of Hsp 70 and old leaf had two times higher TPM of Hsp 90 under 1 h heat stress **[see****Supporting Information—Table S10****]**. We found that genes encoding photosystem II light-harvesting complex protein 2.1 (LHCB2.1), LHCB2.2 and LHCB2.3, a few genes of photosystem I subunits and photosystem II subunits had higher TPM in both young and old leaf under control condition than the heat stress condition **[see****Supporting Information—Table S11****]**. However, after 1 h heat stress the young leaf had double or more TPM transcripts than the old leaf for these genes **[see****Supporting Information—Table S11****]**. Abundance of transcripts for APX and peroxidase superfamily protein did not differ significantly between control and heat stress, while those genes in young leaf had significantly higher expression compared with old leaf **[see****Supporting Information—Table S12****]**. One of the four major genes encoding auxin efflux carrier family protein PIN1, PIN3, PIN4 and PIN7 had decreased TPM after 1 h heat stress in both young and old leaf, while at 10 h heat stress the TPM increased again **[see****Supporting Information—Table S13****]**. And young leaf always had more transcripts expressed than the old leaf in all the conditions. Two genes encoding GA-regulated family protein had significantly greater transcript abundance in young leaf than in old leaf. And under the 1 h heat stress, the expression of gene AT1G74670 (GASA6) in old leaf decreased dramatically, while it was maintained at the similar transcription level upon heat stress in young leaf **[see****Supporting Information—Table S13****]**. Three genes encoding cytokinin response factor (CRF) CRF1, CRF2 and CRF4 had significantly higher expression in young leaf compared with old leaf **[see****Supporting Information—Table S13****]**. The genes encoding ethylene response factor 8 (ERF8), ethylene-responsive element-binding protein (EBP) and ethylene-responsive element-binding factor ERF1 and ERF2 had high expression in old leaf compared with young leaf (Fig. 4; **see****Supporting Information—Table S13**). Overall, genes encoding ABA-responsive elements-binding protein with similarity to transcription factors were highly expressed in old leaf compared with young leaf. Especially, after 1 h heat stress, the old leaf had a significantly greater gene expression than in the young leaf for AT4G34000 gene (i.e. 1108 TPM vs. 199 TPM) **[see****Supporting Information—Table S13****]**. The old leaf expressed greatly higher levels of transcripts than young leaf for genes annotated as calmodulin-binding protein-like protein (*AT2G38800*) and calmodulin-like 41 (*AT3G50770*) suggesting that young and old leaves differ in their signal transduction via Ca^2+^**[see****Supporting Information—Tables 2****and****3****]**.

### Novel genes correlated to young leaf’s heat stress tolerance

Our analyses revealed that functional clusters of 24 and 38 genes involving those annotated as proteins with pectin lyase fold or pectin acetylesterases were expressed at higher levels in young leaves at 0 and 1 h stress conditions, respectively (Tables 2 and 4). Upon 10 h stress, functional clusters involving pectin acetylesterases (Table 6) were higher in the young leaves compared to the old leaf. The major enzymes involved in the pectin degradation are pectin pectylhydrolase, glycanohydrolase and polygalacturonase, in which polygalacturonase was well known to function in fruit ripening, and pollen and abscission zones (Sun and van Nocker 2010; Roongsattham *et al.* 2012).

**Figure 5.**
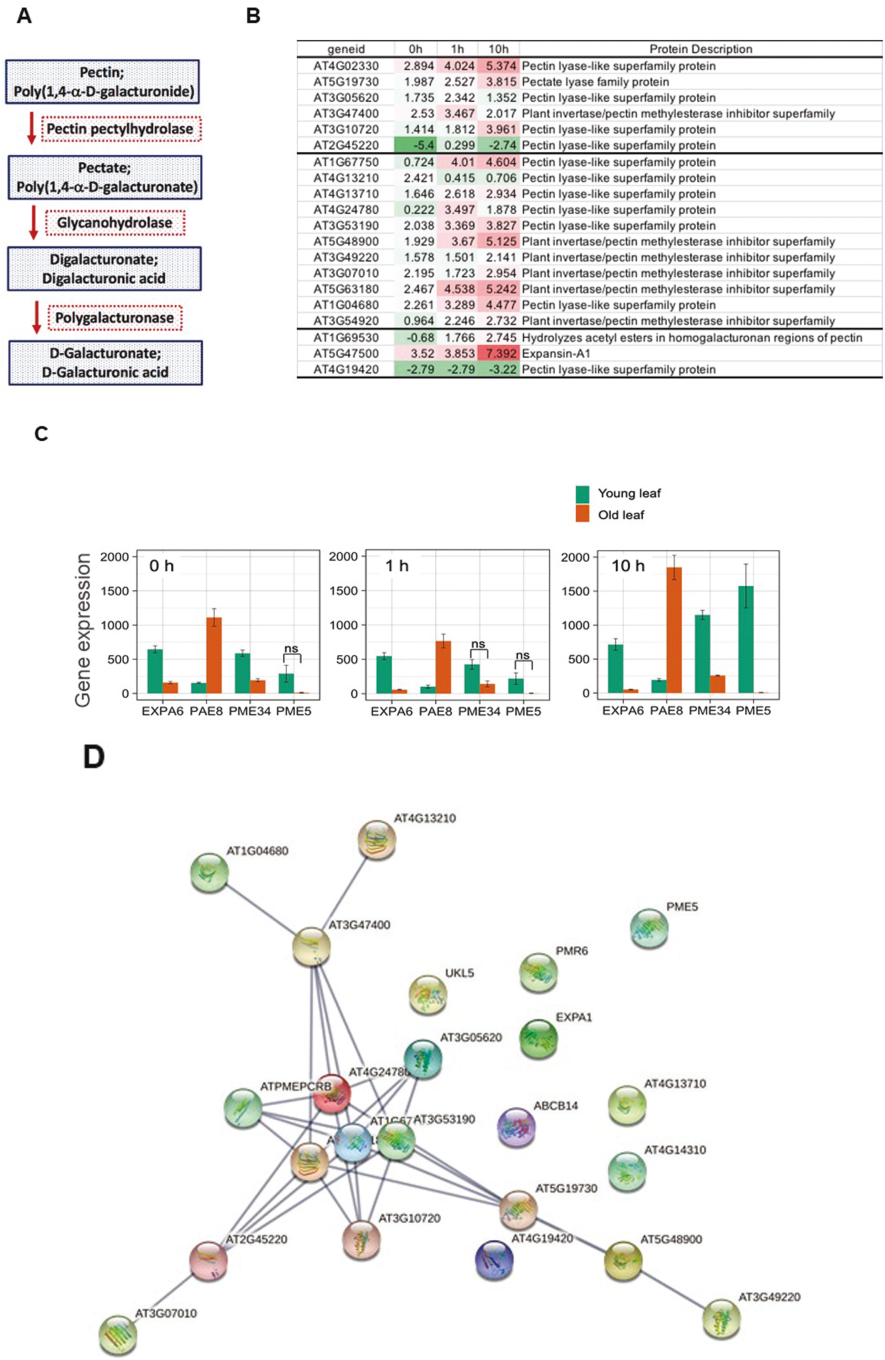
Pectin degradation. (A) The pathway to pectin degradation to D-galacturonic acid. (B) Differentially expressed genes of young leaf compared with old leaf for genes annotated as pectin lyase-like superfamily protein, pectin methylesterase inhibitor superfamily, hydrolases and expansin. Differential transcript levels in a comparison between young and old leaves with red colour indicating higher levels of transcripts in young leaves and green indicating lower levels in young leaves compared to old leaves. (C) Gene expression levels expressed as TPM of genes for AT1G69530 (expansin A1, EXPA1), AT5G47500 (pectin lyase-like superfamily protein, PME5), AT3G49220 (plant invertase/pectin methylesterase inhibitor superfamily, PME34) and AT4G19420 (pectin acetylesterase family protein, PAE8) in young leaf and old leaf under control, 1 h and 10 h heat stress. All comparisons between young and old leaf were significantly (*P* = 0.01) different except three comparisons marked as ns for non-significant. (D) Analysis of genes potentially involved in pectin degradation following a STRING network analysis using a minimum required interaction score of 0.7, line thickness indicating the strength of data support. For colour, please refer to the online images.

The reactions catalysed by pectate lyases, denoted as ‘pectin pectylhydrolase’ and ‘glycanohydrolase’ in Fig. 5A and relative expression levels of genes coding for pectin lyase-like proteins are shown (Fig. 5B). These results are consistent with the hypothesis that young and old leaves differentially regulate pectin lyase-like superfamily proteins in response to the imposed heat stress.

A few genes that were involved in cell wall biosynthesis had significantly different TPM between young and old leaf. The expression levels for genes namely AT1G69530 (expansin A1, EXPA1), AT5G47500 (pectin lyase-like superfamily protein, PME5), AT3G49220 (plant invertase/pectin methylesterase inhibitor superfamily, PME34) and AT4G19420 (pectin acetylesterase family protein, PAE8) are shown in bar diagrams in Fig. 5C. The gene encoding cell wall loosening and stomatal movement EXPA1 had high TPM under control condition in both young and old leaf, while heat stress significantly reduced its gene transcripts (Fig. 5C). Genes involved in biological process of cell wall modification and pectin catabolic process called PME5 and PME34 had higher TPM in young leaf compared with old leaf, and the expression levels were greatly increased under 10 h in young leaf, while not in old leaf (Fig. 5B). In contrast, the gene encoding pectin acetylesterase named PAE8 always had higher expression in old leaf compared with young leaf and its TPM was peaking at 10 h heat stress (Fig. 5C). Network analysis by STRING has grouped several proteins with known interactions including pectin esterase, pectate lyase and hydrolase (Fig. 5D).

## Discussion

Our studies stemmed from our repeated observations that young leaves were damaged significantly less than old leaves for imposed heat stress (Fig. 1). This is in tune with others who observed that young leaves of maize were less affected than older leaves (Karim *et al.* 1999). However, no transcriptomic studies have explored the mechanisms behind this. Previous studies in *A. thaliana* whole plants have recognized multiple transcriptional networks that are induced by the imposed heat stress (Larkindale and Vierling 2008; Yángüez *et al.* 2013; González-Schain *et al.* 2016). While these studies are key to our understanding of heat stress damage and tolerance, they have not focused on potential differential effects between young and old leaves. Others reported that developmentally young and old leaves responded differentially to nitrogen deficiency conditions (Safavi-Rizi *et al.* 2018). In their study leaves of different ages have been shown to have divergent levels of senescence in response to nitrogen deficiency. Young and old leaves can be expected to have differing physiological and developmental status to begin with; however, by identifying gene expression networks that are differentially expressed between young and old leaves upon a precisely imposed heat stress, we aimed to discover genomic mechanisms behind young leaves’ relative tolerance to stress. Differential expression of transcripts could be due to damage to physiological, structural and metabolic processes of the tissue as well as plant’s adaptive responses to the stress. We aimed to identify clusters of gene networks associated with improved heat stress tolerance of young leaves compared to old leaves via clustering genes that are expressed significantly differentially and modulated between young and old leaves (log_2_FC of 5) upon control and heat stress **[see****Supporting Information—Tables S1****–****S14****]**.

Heat stress can damage cellular components through denaturing membranes and proteins by rapid accumulation of ROS (Ohama *et al.* 2016). Based on our results, when *Arabidopsis* Col-0 plants were incubated at high temperature 42 °C, older leaves were damaged significantly more than younger leaves, based on quantitative measures of stress (Fig. 1). For protection from heat stress, it is important for young leaf to rapidly regulate mechanisms to maintain cellular homeostasis. Our analyses of transcripts were consistent with the hypothesis that young leaf’s heat stress tolerance was associated with significantly increased levels of transcripts annotated as chaperones and heat shock proteins **[see****Supporting Information—Table S10****]**. When heat stress occurs, plants detect it rapidly by activating heat shock transcription factors (Hsfs) and enhancing expression of downstream genes which encode heat shock proteins (Hsps). Overexpression of maize HEAT SHOCK FACTOR A2 (ZmHsfA2) in *Arabidopsis* had showed increased expression of *Arabidopsis* RAFFINOSE SYNTHASE, thus increasing the raffinose content and heat tolerance, while overexpression of maize HEAT SHOCK-BINDING PROTEIN 2 (ZmHSBP2) had the opposite regulation of raffinose synthesis (Gu *et al.* 2019). In our RNA-seq results, we also find similar antagonistic regulation between Hsf and HSBP expression and functional clusters marked as chaperonin, DNAJ domain protein and HSP90 binding had higher transcript levels in young leaves compared to old leaves (Table 6). Our results indicated that under 1 h heat stress young leaf had significantly higher transcripts of *HEAT SHOCK-BINDING PROTEIN* genes such as *AT4G02100*, *AT3G10680*, *AT5G20970*, *AT5G04890*, *AT2G27140*, *AT5G51440*, *AT1G20870* and *AT4G10250* and *DNAJ heat shock N-terminal domain-containing protein* genes such as *AT2G05250*, *AT1G77020*, *AT3G47650*, *AT5G03160*, *AT5G27240*, *AT3G51140*, *AT3G62600* and *AT1G75690*, while significantly lower transcript levels for *heat shock transcription factor* genes such as *AT1G67970*, *AT4G18880* and *AT5G62020* compared with old leaf **[see****Supporting Information—Tables 10****and****12****]**.

Pectin as a major component of primary cell wall has been mainly studied in connection with softening of fruits during ripening after harvest (Marín-Rodríguez *et al.* 2002). The pectin methylesterase functions in degrading pectin which is important for plant development and defence against adverse environment. However, knowledge of pectin and pectin methylesterases under heat stress is lacking. Recently, one of the pectin methylesterase genes has been found to contribute to heat stress tolerance (Huang *et al.* 2017). And they suggested that heat stress influenced cell wall plasticity by changing of cell wall metabolism (Y.-C. Huang *et al.* 2017). Our results indicated that genes encoding pectin methylesterase were regulated by heat stress and had differential transcript expression between young and old leaf (Fig. 5C). Gene categories as annotated as PECTATE LYASE-LIKE PROTEIN were expressed significantly differently between young and old leaves under both control and stress conditions, suggesting potentially novel mechanisms of stress tolerance involving cell wall remodelling.

Pectin degradation upon heat stress suggests that the adjustment of cell wall through cell wall loosening and potential stress signalling via metabolites of pectin degradation. This indicated that it was likely that the rapid response of pectin degradation in young leaf compared with old leaf may be a newly identified mechanism to heat stress tolerance.

PME activity is a critical determinant of plant response to high temperature (Wu *et al.* 2018). PME5 has been reported to be regulated by phyllotaxis transcription factor BELLRINGER (BLR) which indicated PME is involved in organ initiation and cell formation as an important regulatory mechanism (Peaucelle *et al.* 2011). The gene encoding BLR (such as *AT5G02030*) was not expressed differentially between young and old leaf while PME5 showed significant differential expression especially under 10 h heat stress. Our results showed that three genes *AT3G14310*, *AT4G33220* and *AT3G59010* encoding PME3, PME44 and PME61 had higher transcript expression in young leaf than old leaf. PME34 had been reported to function in regulating guard cell wall flexibility and mediate heat response (Huang *et al.* 2017). Conversely, PAE8 and PAE9 which have pectin acetylesterase activity and function together to remove one-third of cell wall acetate (de Souza *et al.* 2014) had higher expression in old leaf.

Contrasting high temperature greatly inducing *PME* in young leaf, ABA-responsive elements-binding factor *ABF3* and *ABF1*, and actin-depolymerizing factors (*ADF5*) which promotes stomatal closure (Qian *et al.* 2019) had higher expression in old leaf when exposed to heat stress. Our results suggest that pectin degradation, ABA-related processes and heat shock protein signalling cascades play roles in the differential tolerance of young leaves to heat stress. Hence, genes involved in these processes could be explored in future studies to find candidate genes for improving heat stress tolerance of crops.

## Conclusions

- When the model plant *Arabidopsis* wild-type Col-0 plants were incubated under heat stress, old leaves were damaged significantly more than young leaves based on quantitative measures of plant stress.
- To test the hypothesis that differential gene expression could reveal the mechanisms for young leaf’ relative tolerance to stress, we conducted an RNA-seq study of young and old leaves of plants exposed to control and stress conditions for 1 h and 10 h. Results indicated young leaf had significantly differential expression of multiple genes under stress compared to the old leaf.
- Under 1 h heat stress, young leaf has showed upregulation of a few heat stress-related downstream transcriptional cascades, including *HEAT SHOCK PROTEINS, HEAT SHOCK TRANSCRIPTION FACTOR* and *DNAJ HEAT SHOCK PROTEIN*.
- Under 10 h heat stress, genes annotated as *PECTATE LYASE-LIKE PTOTEIN* were expressed significantly higher in young leaf, while genes encoding PME5 and PME34 had higher expression in old leaf under heat stress suggesting that pectin degradation may be a key factor distinguishing young and old leaves in their tolerance to heat stress.
- Differential expression of genes involved in cutin and wax biosynthesis, pentose and glucuronate interconversions, cellulose degradation, indole glucosinolate metabolism and RNA splicing suggests potential gene clusters for consideration in future studies on plant tolerance to heat stress.

## Supporting Information

The following additional information is available in the online version of this article—

Table S1. A comparison of transcript levels between young leaf and old leaf at control temperature.

Table S2. A comparison of transcript levels between young leaf and old leaf following 1 h of heat stress.

Table S3. A comparison of transcript levels between young leaf and old leaf following 10 h of heat stress.

Table S4. A comparison of transcript levels between young leaf after 1 h stress and young leaf at control condition.

Table S5. A comparison of transcript levels between young leaf after 10 h heat stress and young leaf after 1 h of heat stress.

Table S6. A comparison of transcript levels between young leaf after 10 h of heat stress and young leaf under control condition.

Table S7. A comparison of transcript levels between old leaf after 1 h of heat stress and old leaf under control condition.

Table S8. A comparison of transcript levels between old leaf after 10 h heat stress and old leaf after 1 h of heat stress.

Table S9. A comparison of transcript levels between old leaf after 10 h of heat stress and old leaf under control condition.

Table S10. Transcript levels of genes differentially expressed between young and old leaves annotated as chaperones, heat shock proteins or heat shock transcription factors.

Table S11. Transcript levels of genes differentially expressed between young and old leaves annotated as proteins involved in photosynthesis and photorespiration.

Table S12. Transcript levels of genes differentially expressed between young and old leaves annotated as coding for proteins involved in oxidative stress.

Table S13. Transcript levels of genes differentially expressed between young and old leaves annotated as coding for proteins involved in phytohormone-related pathways.

Table S14. Transcript levels of genes differentially expressed between young and old leaves annotated as cell wall remodelling processes.

plac024_suppl_Supplementary_Tables_S1_S9

plac024_suppl_Supplementary_Tables_S10_S14

## Data Availability

All data are available in **Supporting Information—Tables S1**–**S14**.

## Notes

**Phenome, Genome & Environment. Chief Editor**: Colleen Doherty
